# Relationship Between Skin Scales and the Main Flow Field Around the Shortfin Mako Shark *Isurus oxyrinchus*

**DOI:** 10.3389/fbioe.2022.742437

**Published:** 2022-04-25

**Authors:** Chengchun Zhang, Meihong Gao, Guangyuan Liu, Yihua Zheng, Chen Xue, Chun Shen

**Affiliations:** ^1^ Key Laboratory of Bionic Engineering (Ministry of Education), Jilin University, Changchun, China; ^2^ State Key Laboratory of Automotive Simulation and Control, Jilin University, Changchun, China; ^3^ Weihai Institute of Bionics, Jilin University, Weihai, China; ^4^ China Aerodynamics Research and Development Center, Mianyang, China; ^5^ Advanced Materials Industry Institute of Guangxi Academy of Science, Nanning, China; ^6^ School of Energy Science and Engineering, Harbin Institute of Technology, Harbin, China

**Keywords:** biomimetics, shark, main flow field, scale, relationship

## Abstract

The aim of this study was to reveal potential relationship between the main flow field around a shortfin mako shark and the surface morphology of shark skin. Firstly, a numerical simulation using the large eddy simulation (LES) method was conducted to obtain the main flow field around a smooth shark model. Then, the surface morphology characteristics of a shark (*Isurus oxyrinchus*) at different positions were characterized by scanning electron microscope (SEM), which showed that the morphology, riblet size, and density of scales at different positions on the shark were significantly different. At positions where the surfaces face into the water flow direction (i.e., nose and leading edge of fins), the scales were flat and round, with a lower density, and the pressure or wall shear stress (WSS) was greater. Scales with three longitudinal riblets ending in three tips were found on the middle and trailing edges of the first dorsal fin and caudal fin, where water flow states progress from transitional to turbulent. The ranges of the ratio of riblet depth to spacing (RD/RS) in the anterior zone, middle zone and posterior zone of the shark were 0.05–0.17, 0.08–0.23, and 0.32–0.33, respectively. The riblet angle generally followed the flow direction, but it varied across different areas of the body. The turbulence intensity increased gradually across the first dorsal fin, pectoral fin, caudal fin, and the shark body overall. In summary, it was found that the microstructure riblets on the shark skin surface, generally thought to be drag reduction structures, were only located in transitional and turbulent regions at the middle and trailing edge of the shark body and fin surfaces, and there were almost no microstructural grooves in the laminar flow regions along the leading edge. These findings can provide design guidance for engineering applications of bionic riblet surfaces. Riblets placed in transitional and fully turbulent regions can be used to effectively reduce drag. The riblet direction should be consistent with the direction of flow.

## Introduction

The adaptive morphological and behavioral characteristics of large aquatic organisms in marine environments have evolved over many generations, most commonly towards reducing total drag (Fish, 1998). Certain sharks are among the fastest fishes in the ocean. In particular, the shortfin mako shark (*Isurus oxyrinchus*) is able to reach swimming speeds of up to 70 km h^−1^ when feeding (Compagno et al., 2005). Its speed is not only determined by its muscle capacity and shape, but also the ultrastructure of the surface of its skin (Bernal et al., 2003). Denticles of mako shark skin play a similar role as vortex generators and changes in their size, shape, and direction can alter the boundary layer, thereby reducing resistance (Ott et al., 2020).

Drag reduction mechanisms and technical research of the shark skin riblet structure have been intensively studied for several decades. Two drag reduction mechanisms have been recognized in shark skin. The first is that riblets can reduce momentum transfer and shear stress by preventing cross-flow over the scales (Bechert et al., 2000b). The second is that the scales are flexible and capable of bristling to stop reversed flow across the skin surface (Lang et al., 2011; Du Clos et al., 2018; Santos et al., 2021). NASA Langley Research Center (Walsh and Weinstein, 1978) has carried out research on the drag reduction performance of ribbed surfaces. Their experimental results showed that the drag reduction ratio of a longitudinal V-groove surface could reach 8% at low speeds (Walsh, 1982), and the dimensionless size of the groove with the drag reduction effect was given in a follow-up study (Walsh, 1983). After German researchers (Reif and Dinkelacker, 1982; Bechert and Bartenwerfer, 1989) described riblet scales on fast swimming sharks (*Isurus oxyrinchus, Carcharhinus faleiformis*), drag reduction research inspired by shark skin have spiked the interest of the scientific community. Early research showed that a 3D ribbed surface on a flat plate could achieve a drag reduction of about 6%. Further research based on this observation indicated that a 3D fin-shaped surface imitating the scales of the mako shark and great white shark could achieve drag reduction up to 7.3% (Bechert et al., 2000a). Because the riblet surface does not require additional energy consumption and has a very simple structure, it is considered to be the most potent turbulence drag reduction method.

The optimal riblet geometries and dimensions based on fluid-flow characteristics have been investigated by many scholars. The ideal geometry of h/s (the ratio of riblet height to spacing) was about 0.5 for blade riblets with a no-slip condition, with drag reduction rates reaching nearly 10%. The maximum drag reduction rate by scalloped riblets and sawtooth riblets was about 6% at h/s ∼ 0.7 and 5% at *α* ∼ 60°, respectively (Bechert et al., 1997). With the development of advanced manufacturing technology, researchers have produced bionic surfaces that are similar to shark skin and even have been able to replicate scales, and the drag reduction performance was further confirmed (Luo et al., 2016). Bionic surfaces of bonnethead shark skin covered with microstructures were prepared using a 3D printing method, and the drag reduction rate in low-speed water was 8.7% (Wen et al., 2014). The shark (*Carcharhinus brachyurous*) skin surface manufactured by researchers (Chen et al., 2015) based on the UV curing shrinkage method reduced drag by up to 11%. The surface of shark (*Isurus oxyrinchus*) skin made using the synthetic biological replication molding method had a drag reduction rate of 24.6% at a water velocity of 8 m/s (Zhang et al., 2011).

Although many studies have shown that microstructure surfaces inspired by shark skin can reduce surface frictional resistance caused by fluid viscosity (Dean and Bhushan, 2010; Martin and Bhushan, 2014; Bai et al., 2016; Boomsma and Sotiropoulos, 2016; Heidarian et al., 2018), this drag reduction method using microstructure surface is rarely applied in engineering. In addition to the high cost of processing and manufacturing, accurately arranging the microstructures on working parts has proven challenging in engineering applications. For instance, it is often difficult to obtain a satisfactory drag reduction effect when utilizing the same microstructure all over the working surface of a machine, such as submarine or cruise missile. Microstructures on different surfaces should be designed with different sizes and morphologies. In the long-term evolution process, sharks have evolved suitable surfaces for their flow field. Scales in different parts of the body show different morphological characteristics, which minimize resistance over the body (Raschi and Tabit, 1992; Lauder and Di Santo, 2015; Dillon et al., 2017; Ankhelyi et al., 2018; Rangel et al., 2019). In other words, the morphology and size of the scales are related to flow field, and investigating this hypothesis will be highly useful from the perspective of bionics. Therefore, this study investigated the relationship between the morphology, riblet size, and density of scales and the flow field surrounding the shortfin mako shark.

## Materials and Methods

### Creating the Smooth Shark Model

**FIGURE 1:**
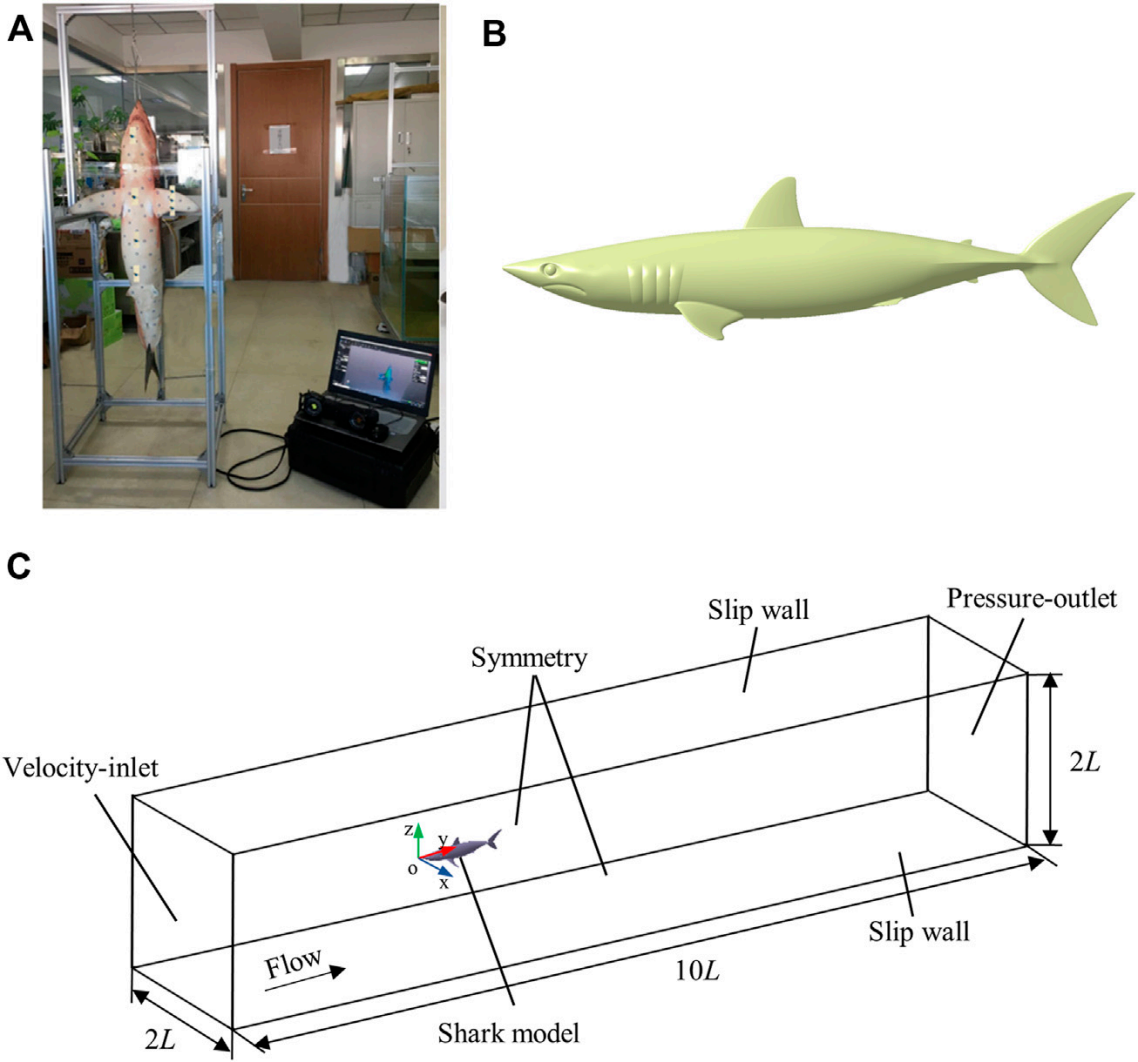
3D smooth shark model. **(A)** 3D scanning of a shark, **(B)** The three-dimensional smooth geometric shark model. **(C)** Schematic of the computational domain and boundary conditions.

A shortfin mako shark (*Isurus oxyrinchus*) (number of permit: SY202104051) 1.32 m in total length (*L*) was scanned using a hand-held Laser Scanner HandySCAN BLACK Elite at a resolution of 0.025 mm. In this study, the pectoral fins, first dorsal fin, and caudal fin were fixed within the scanning frame using threadlets to ensure the correct cruising attitude of the shark in the ocean (Figure 1A). A 3D reconstruction of the shark in STL format was used to characterize the main flow field around the shortfin mako shark (Figure 1B).

### Computational Domain and Boundary Conditions

The computational domain of the smooth shark model should avoid the negative effects of boundary conditions. To ensure this, the computational domain of smooth shark model was set to 10 *L* × 2 *L* × 2 *L*. The tip of the shark’s nose was set as the origin of the coordinate system and the x, y, and *z* axes denoted the spanwise, streamwise, and wall-normal, respectively (Figure 1C). The inlet boundary was set 3 *L* from the tip of the shark’s nose and the outlet boundary was placed 6 *L* downstream of the posterior end of the caudal fin. The perpendicular distance between the wall and the origin of the coordinate system was *L*. The inlet boundary condition was specified as having a uniform velocity and the outlet boundary condition was set as pressure. The side walls were prescribed to have symmetrical conditions. Both top and bottom walls were set to a shear stress of 0. The Reynolds number was calculated according to *R*_*e*_ = (*ρVL*) *μ* ^*−1*^ = 4.5 × 10^6^, where *ρ* is the density of seawater (1030 kg m^−3^), *V* is the flow velocity of seawater (5 m s^−1^), and *μ* is the dynamic viscosity of seawater (1.52 × 10^–3^ kg m^−1^ s^−1^).

### Solving Methods for Flow Simulation

The simulation was started by running a steady-state simulation with a standard k-ε turbulence model to generate more realistic turbulence statistics for LES (Russo and Basse, 2016). To capture the unsteady flow within the turbulent boundary layer, the Smagorinsky-Lilly subgrid-scale turbulence model was adopted in the LES. The transient calculation results using the LES turbulence model have been shown to be close to experimental pressure distribution measurements (Andrus and Post, 2005). The gradients of the solution variables were computed using the Green-Gauss Node based gradient evaluation. The second order scheme and bounded central differencing scheme were used to deal with the spatial discretization of pressure and momentum. The Pressure-Implicit with Splitting of Operators (PISO) pressure-velocity coupling scheme was employed to derive equations for pressure from the momentum equations and the continuity equation.

### Grid Independence and Accuracy Tests

Tetrahedral grids were employed to discretize the computational domain. The number of grids affect the computational efficiency and simulation accuracy, so it needs to be considered conducting grid accuracy tests. Systematically refined grid schemes with constant refinement ratios of $\sqrt{2}$ were applied to estimate the errors, as recommended by Wilson (Wilson et al., 2001). The details of the three grid schemes were assessed, including the total number of elements, the distance to the nearest wall, quality, max aspect ratio, non-orthogonality, and max skewness (Table 1). The non-dimensional wall distance *y* ^*+*^ was 4 in this article, which met the requirements of the numerical calculations. A grid independence study was performed on the smooth shark model by comparing the surface pressure coefficients of the three different grid schemes. The surface pressure coefficient (*C*_*p*_) of the shark is defined by

$$C_{P}=\frac{P-P_{ref}}{\left(\rho V^{2}/2\right)}$$ (1)

where *p* is the static pressure at the point at which surface pressure coefficient is being evaluated and *P*_*ref*_ is the static pressure in the freestream.

**TABLE 1:** The study of grid independence.

Details of Grid	Grid Schemes		
	Grid1	Grid2	Grid3
Total number of elements	5412905	15156135	43952792
Distance to the nearest wall, *y*_*0*_ (mm)	0.031	0.031	0.031
Quality	0.2	0.2	0.2
Max aspect ratio	26.81	19.24	13.46
Non-orthogonality	13–62	14–62	14–62
Max skewness	2.64	2.05	1.98

**FIGURE 2:**
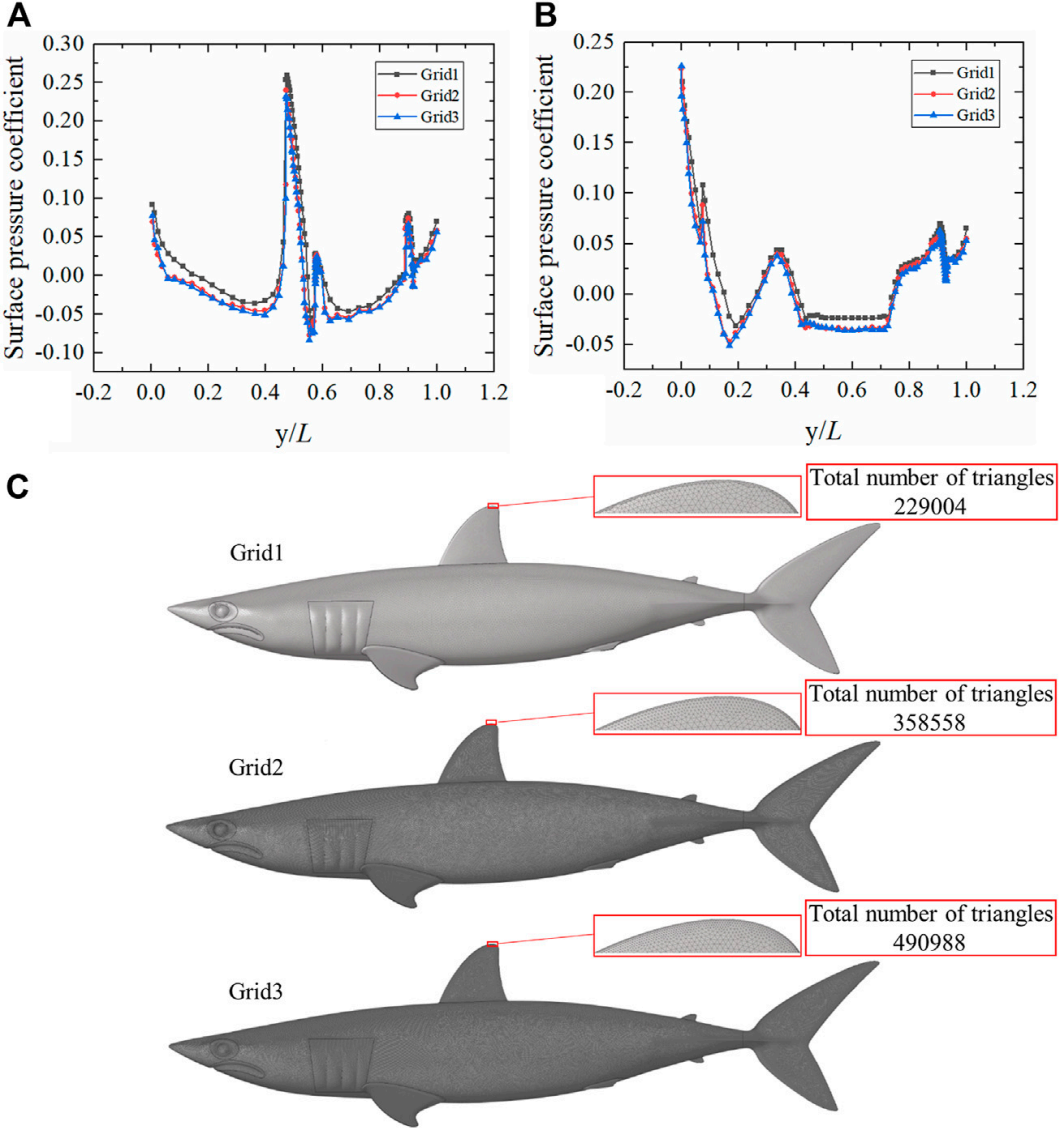
Grid independence analysis of smooth shark model. **(A)** Comparison of surface pressure coefficients on the upper surface of the smooth shark model for the three grid schemes. **(B)** Comparison of surface pressure coefficients on the lower surface of the smooth shark model for the three grid schemes. y/L is the ratio of the position coordinate to the length of the shark. **(C)** Smooth shark model surface grids. The total number of triangles are displayed in the upper right corner.

Comparisons of surface pressure coefficients from the three different grid schemes are presented in Figures 2A,B (Supplementary Table 1). The surface pressure coefficient calculated using Grid1 was much larger than those when using Grid2 and Grid3, while the surface pressure coefficient of Grid2 was basically coincident with Grid3. Because of the huge computational expense required when using Grid3, Grid2 was adopted to calculate the flow field of the shortfin mako shark in this study. The three grid schemes of the shark model are displayed in Figure 2C.

### Collecting and Processing the Shark Skin Samples

**FIGURE 3:**
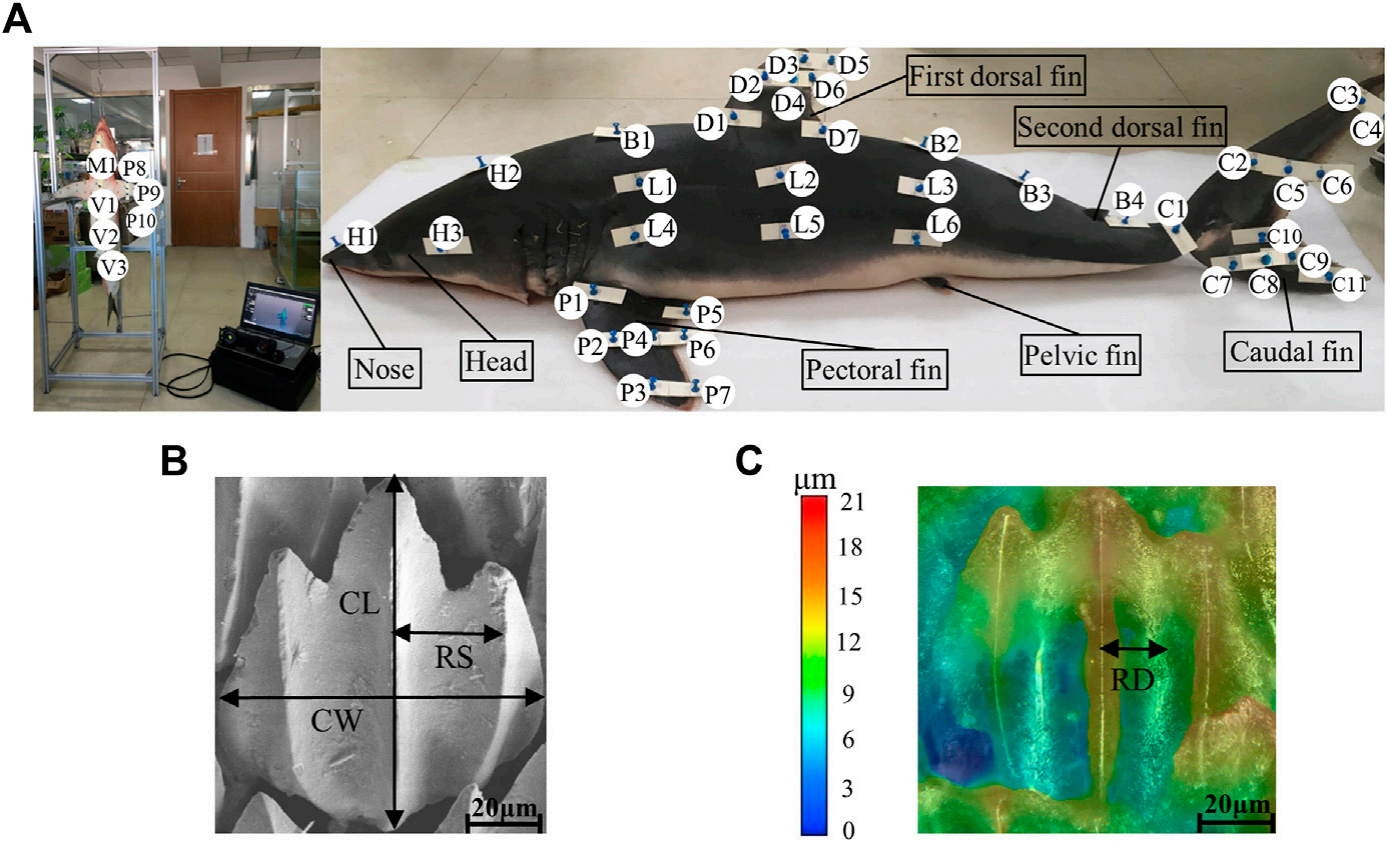
Collection and measurement of shortfin mako shark samples. **(A)** Locations of collected samples on the shortfin mako shark. Each letter represents a sampling region: H, head; B, back; D, dorsal; C, caudal; L, lateral; P, pectoral; V, ventral; and M, lower jaw. **(B)** Measurements of crown length and crown width were taken from the medial riblet. Riblet spacing was measured from the groove adjacent to the central riblet. CL, crown length; CW, crown width; and RS, riblet spacing. **(C)** Riblet depth was measured from the groove adjacent to the central riblet. RD, riblet depth. The color gradient corresponds to riblet depth.

In order to explore the relationship between the flow field around the shark and the adaptive growth of scales, the physical parameters of scales were measured. The morphology, riblet size, roughness, and composition of shark scales have been previously studied in fine detail (Motta et al., 2012; Popp et al., 2020; de Lima Viliod et al., 2021). A sub-set of 45 skin samples of 2 cm × 2 cm were carefully dissected from the different parts of the shark using pre-made templates consisting of plastic substrates and pins (Figure 3A). Because the pins can damage scales, the samples were collected 1 cm horizontally inside of the pins.

Collected samples were fixed on numbered plastic substrates with pins according to the sequence of markings on the shark. The samples were soaked in formalin to prevent them from spoiling and decaying at room temperature. Furthermore, to maintain the original structure and mechanical strength of the shark skin, the samples were processed as follows. First, an ultrasonic cleaner was used to rinse blood and shredded meat from the shark skin (40 min). Samples were then fixed with 2.5% glutaraldehyde (8 h, 4°C) and rinsed (4 × 15 min) in phosphate buffered saline (0.1 mol/L, pH = 7.2, room temperature). Following this, the samples were dehydrated in gradually increasing concentrations of ethanol (30, 50, 75, 80, 95, and 100%, 20 min each), then immersed in a mixture of isopentyl acetate and ethanol (1:1, 15 min) and dipped in 100% isopentyl acetate for 10 min. Finally, the samples were placed on culture dishes and dried under a vacuum (Supplementary Image 1).

### Measurements of Morphology, Riblet Size, Angle, and Density on Scales of Shortfin Mako Shark

Morphology, riblet size, angle, and density are important parameters for characterizing shark skin scales. In this study, the riblet sizes of scales were quantified by measuring crown length (CL), crown width (CW), riblet depth (RD), and riblet spacing (RS) (Figures 3B,C **)**. Because the gold-spray treatment increases the surface thickness of samples, the RD and RS were recorded first to minimize measurement error. RD and RS of 50 scales per micrograph were obtained with a digital microscope (KEYENCE VHX-6000). The samples were then coated with gold in an argon atmosphere. The morphologies of the scales were characterized using SEM (Zeiss 710) at an accelerating voltage of 20 kV, working distance of 8.5 mm, and magnification of ×100. The riblet angle of each skin sample relative to the flow direction was obtained from 20 randomly selected scales. Because the riblet angle of a scale is measured in a 2D plane, SEM images do not fully express the spatial three-dimensional state of shark skin scales. Ten regions with areas of 1 mm^2^ were randomly selected in each sample to obtain the mean ± S.D. of the density (number of scales, mm^−2^). Five micrographs were taken per sample and 10 scales from each were measured to calculate CL and CW (total N ≥ 50 scales per sample).

## Results

### Analysis of Morphology, Size, and Density of Shark Skin Scales

**FIGURE 4:**
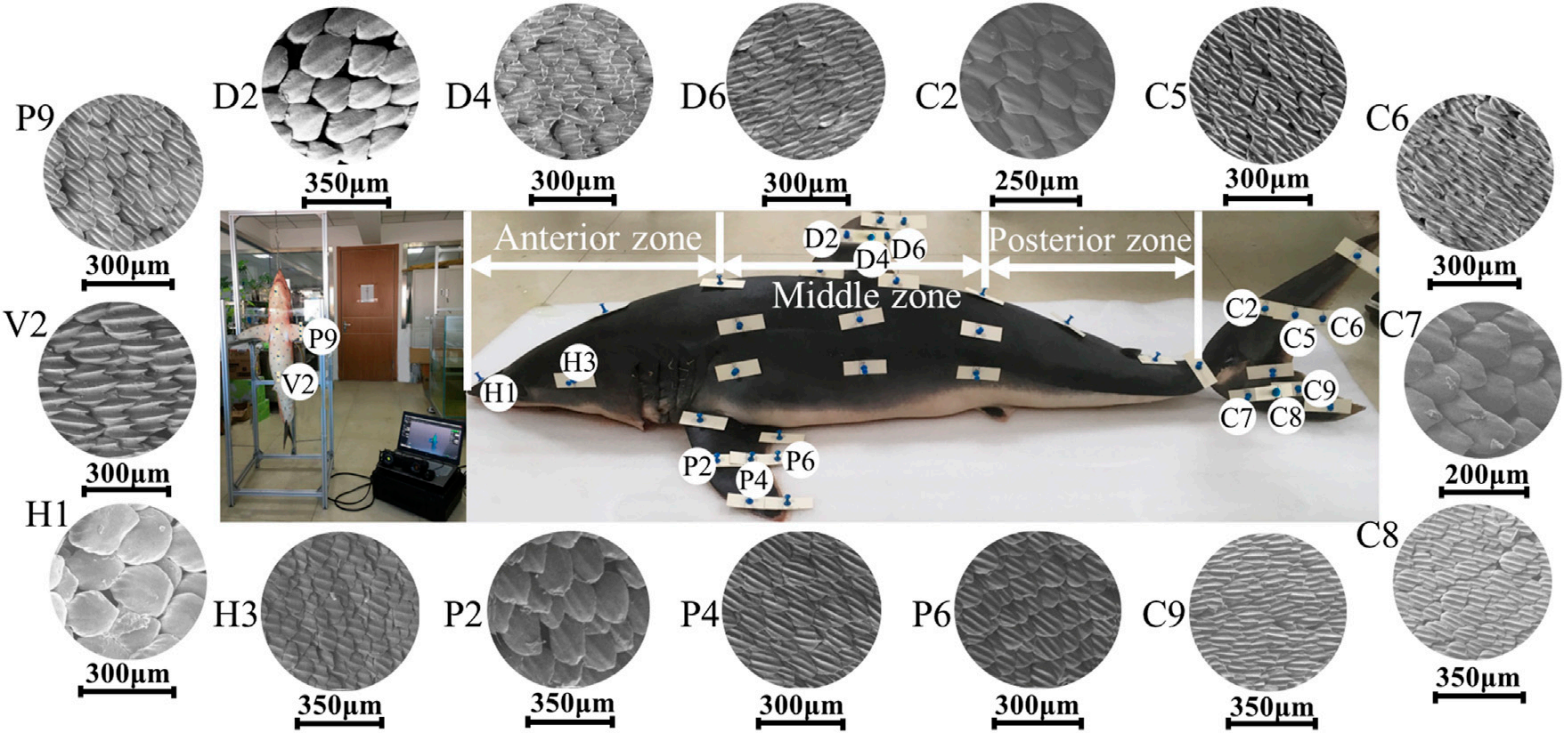
Micrographs (SEM) showing the morphology of scales in shark skin samples.

This study clearly illustrated the great differences in the morphology, riblet size, and density of scales among surfaces on the shortfin mako shark. The majority of the scales from the measured samples of the shark had three longitudinal riblets, but the scales on the nose and leading edge of fins were flat and round, as shown in Figure 4. The parameters of the scales on the 45 samples are given in Table 2. The maximum CL (286 ± 25 μm) and CW (255 ± 22 μm) values were found on the leading edge of the pectoral fin (P2), and the minimum CL (105 ± 7 μm) and CW (87 ± 4 μm) values were measured on the trailing edge of the caudal fin (C11). The CL and CW of the scales gradually decreased from the leading edge, through the middle, and to the trailing edge of the fins. The ratio of crown length to width (CL/CW) ranged from 1.02 to 1.45. The CL/CW was generally larger on ventral regions (V1, V2, and V3), which indicated that these regions had longer and narrower scales.

**TABLE 2:** Morphometric measurements from scales of shortfin mako shark.

Region	H1	H2	H3	B1	B2	B3	B4	C1	C2	C3	C4	C5	C6	C7	C8
CL(μm)	239 ± 23	215 ± 21	186 ± 17	225 ± 22	201 ± 18	191 ± 14	138 ± 12	244 ± 26	221 ± 23	129 ± 10	121 ± 8	165 ± 15	198 ± 15	159 ± 11	125 ± 10
CW(μm)	207 ± 13	173 ± 16	174 ± 15	178 ± 17	177 ± 16	169 ± 13	102 ± 7	184 ± 12	173 ± 14	102 ± 6	93 ± 5	144 ± 11	148 ± 10	129 ± 10	93 ± 9
RD(μm)	0 ± 0	2 ± 0	7 ± 0	3 ± 0	9 ± 1	10 ± 1	9 ± 1	0 ± 0	0 ± 0	0 ± 0	8 ± 0	8 ± 1	9 ± 0	0 ± 0	7 ± 0
RS(μm)	N/A	38 ± 5	41 ± 6	39 ± 5	41 ± 6	31 ± 4	27 ± 2	N/A	N/A	N/A	28 ± 2	34 ± 4	27 ± 2	N/A	32 ± 4
CL/CW	1.15	1.24	1.07	1.26	1.14	1.13	1.35	1.33	1.28	1.26	1.3	1.15	1.34	1.23	1.34
RD/RS	N/A	0.05	0.17	0.08	0.22	0.32	0.33	N/A	N/A	N/A	0.29	0.24	0.33	N/A	0.22
Density	52 ± 3	59 ± 6	58 ± 6	61 ± 7	64 ± 8	78 ± 9	65 ± 7	41 ± 2	47 ± 3	54 ± 3	56 ± 4	52 ± 3	48 ± 2	49 ± 3	53 ± 5
Region	C9	C10	C11	D1	D2	D3	D4	D5	D6	D7	L1	L2	L3	L4	L5
CL(μm)	147 ± 11	188 ± 16	105 ± 7	204 ± 19	192 ± 13	163 ± 16	137 ± 12	138 ± 11	145 ± 15	153 ± 16	182 ± 18	218 ± 21	227 ± 23	138 ± 8	179 ± 16
CW(μm)	112 ± 6	151 ± 13	87 ± 4	155 ± 12	151 ± 11	126 ± 12	104 ± 7	113 ± 11	124 ± 12	125 ± 13	162 ± 13	175 ± 15	209 ± 20	135 ± 14	153 ± 14
RD(μm)	8 ± 0	10 ± 1	9 ± 1	0 ± 0	0 ± 0	0 ± 0	3 ± 0	5 ± 0	4 ± 0	3 ± 0	6 ± 0	9 ± 0	8 ± 0	8 ± 1	7 ± 0
RS(μm)	30 ± 4	29 ± 2	30 ± 3	N/A	N/A	N/A	25 ± 2	21 ± 2	20 ± 2	24 ± 3	41 ± 6	40 ± 5	43 ± 6	39 ± 4	35 ± 3
CL/CW	1.31	1.25	1.21	1.32	1.27	1.29	1.32	1.22	1.17	1.22	1.12	1.25	1.09	1.02	1.17
RD/RS	0.27	0.34	0.3	N/A	N/A	N/A	0.12	0.24	0.2	0.13	0.15	0.23	0.19	0.21	0.2
Density	55 ± 6	48 ± 4	59 ± 5	41 ± 2	44 ± 2	47 ± 3	52 ± 4	51 ± 3	49 ± 3	48 ± 3	47 ± 2	44 ± 2	41 ± 1	51 ± 5	49 ± 3
Region	L6	P1	P2	P3	P4	P5	P6	P7	P8	P9	P10	M1	V1	V2	V3
CL(μm)	211 ± 16	273 ± 24	286 ± 25	221 ± 21	195 ± 19	191 ± 14	215 ± 15	124 ± 13	173 ± 16	213 ± 15	195 ± 13	161 ± 16	194 ± 21	228 ± 23	206 ± 19
CW(μm)	169 ± 12	245 ± 21	255 ± 22	187 ± 14	161 ± 13	172 ± 10	179 ± 9	115 ± 7	147 ± 11	175 ± 10	166 ± 6	130 ± 11	134 ± 12	168 ± 14	143 ± 12
RD(μm)	7 ± 0	0 ± 0	0 ± 0	0 ± 0	6 ± 0	3 ± 0	4 ± 0	5 ± 0	0 ± 0	6 ± 0	7 ± 0	6 ± 0	12 ± 2	11 ± 2	13 ± 2
RS(μm)	39 ± 4	N/A	N/A	N/A	34 ± 3	29 ± 2	33 ± 3	36 ± 4	N/A	26 ± 2	29 ± 3	24 ± 2	33 ± 4	42 ± 6	40 ± 5
CL/CW	1.25	1.11	1.12	1.18	1.21	1.11	1.2	1.08	1.18	1.22	1.17	1.24	1.45	1.36	1.44
RD/RS	0.18	N/A	N/A	N/A	0.18	0.1	0.12	0.14	N/A	0.23	0.24	0.25	0.36	0.26	0.33
Density	45 ± 2	42 ± 1	40 ± 1	44 ± 2	47 ± 3	49 ± 4	48 ± 3	59 ± 6	57 ± 6	47 ± 3	51 ± 5	56 ± 7	54 ± 5	45 ± 5	50 ± 3

Sampling regions are displayed in Figure 3. Values are means ± SD (Standard Deviation). When the standard deviation is less than 0.5, it is recorded as 0. The ratio of riblet length-width and depth-spacing is the ratio of two mean values and there has no SD. N/A means that the scales are smooth without riblets.

The values of the riblet RD in skin samples ranged from 2 ± 0 to 13 ± 2 μm. The riblet RS ranged from 20 ± 2 μm on the trailing edge of the first dorsal fin (D6) to 43 ± 6 μm on the lateral surface (L3). The range of the ratio of riblet depth to spacing (RD/RS) was 0.05–0.36. The RD/RS on the leading edge of the first dorsal fin and caudal fin was nearly zero (smooth scales), and increased progressively from the middle to the trailing edge. In the pectoral fins, the larger values of RD/RS were found in the middle and trailing edge of the ventral surface. The riblets RD/RS of the shark body increased gradually from the anterior zone, the middle zone, and to the posterior zone, with values of 0.05–0.17, 0.08–0.23, 0.32–0.33, respectively.

The higher densities of scales were found on the back regions (B1, B2, B3, and B4) of the shark. The densities of scales on the trailing edge of the first dorsal fin were higher than the leading edge. Interestingly, the scale densities on the dorsal side of the pectoral fin progressively increased from the leading edge, the middle, and to the trailing edge. However, the highest scale density on the ventral side of the pectoral fin was recorded at the leading edge. On the caudal fin, the highest density was found on the lateral surfaces of the dorsal and ventral lobes, and the scales on the trailing edge were denser than on the leading edge.

### Relationship Between the Main Flow Field Around the Shortfin Mako Shark and Its Skin Scales

**FIGURE 5:**
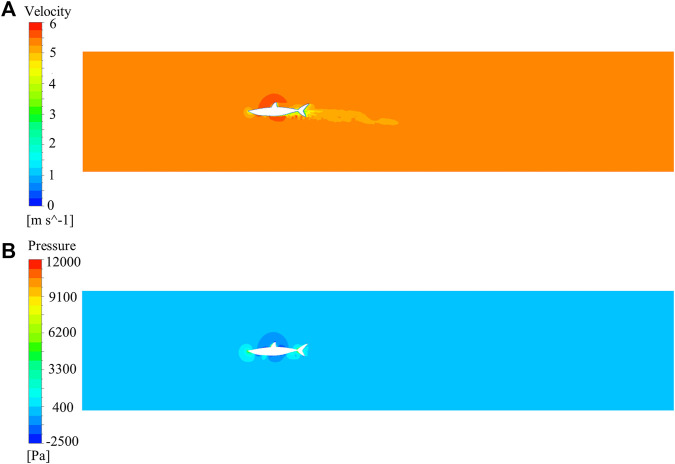
Contours for the whole computational domain of the shark model. **(A)** Velocity contour, **(B)** Pressure contour.

**FIGURE 6:**
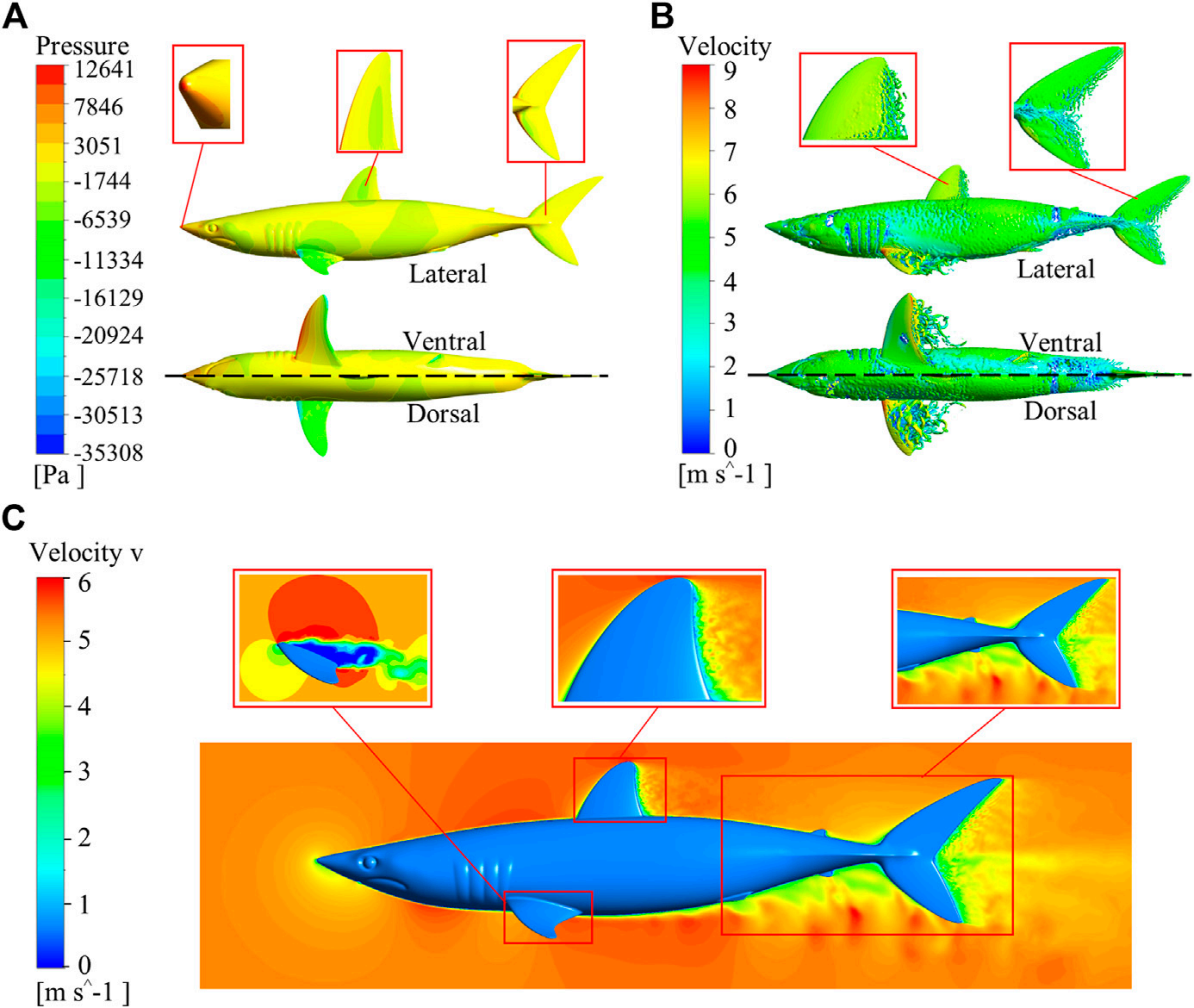
Distributions of pressure, vorticity, and velocity (v component) around the smooth shark model. **(A)** Pressure distribution, **(B)** Vorticity distribution; lateral view (upper) and ventral, dorsal views (lower). The inset magnified portions are the nose, first dorsal fin, and caudal fin. **(C)** Distribution of velocity (v component). The enlarged parts are the pectoral fin, first dorsal fin, posterior zone of the shark body, and caudal fin.

The Ansys Fluent software was used to carry out all the simulations in this study. The flow field of the shark at a 0° angle of attack was calculated. Velocity and pressure contours for the whole computational domain of the shark model are shown in Figure 5. It can be seen that the domain size was large enough to avoid wall effects. The morphology and size of shark skin scales must be adapted to the surrounding flow field. However, calculating the flow field around a shark with scales was nearly impossible. The microstructures on the shark skin were small enough relative to the macro body, which should have the quantitative effect on the boundary layer flow field. This relatively small quantitative effect would not remarkably change the qualitative distribution regularity of parameters in the macro main flow field, such as the turbulence intensity, pressure, etc. Based on this assumption, the smooth shark model was chosen to study the relationship between the parameters of the macro main flow field and the distribution law of the collected shark scales. The distributions of pressure, vorticity, and velocity (v component) of the smooth shark model are presented in Figure 6. The regions of the shortfin mako shark experiencing the higher pressure were those facing the water flow (nose, leading edges of dorsal and caudal fins, and ventral sides of the pectoral fins), where the densities of the scales were generally lower and the scales were generally smooth (Figure 6A). There were higher density scales with riblets on the middle and trailing edge of the first dorsal fin and caudal fin, where the pressure was relatively low.

The flow states along the first dorsal fin and caudal fin progressed from laminar, transitional, to turbulent (Figure 6B **)**. Correspondingly, the RD/RS of the scales gradually increased along the laminar-turbulent zone. The distributions of vortices were orderly and obviously, and the main flow field exhibited a laminar regime at the nose and the leading edges of fins. The vortices of the trailing edge of the first dorsal fin and caudal fin were complicated and disorderly, frequently departing from the surface. The values of RD/RS in the first dorsal fin (D5) and caudal fin (C10) were 0.24 and 0.34, respectively. It can be seen that the maximum values of RD/RS on the first dorsal fin and caudal fin were located in the fully developed turbulent region. On the pectoral fins, the laminar-turbulent transition zone occurred closer to the leading edge and the largest RD/RS value was recorded at the trailing edge on the ventral side. Flow separation was obvious at the trailing edge of the first dorsal fin, caudal fin and the posterior zone of the body (Figure 6C). The scales at these regions had riblet structures, and their RD/RS values and densities were larger. The flow separation on the pectoral fin occurred earlier than first dorsal fin and caudal fin.

**FIGURE 7:**
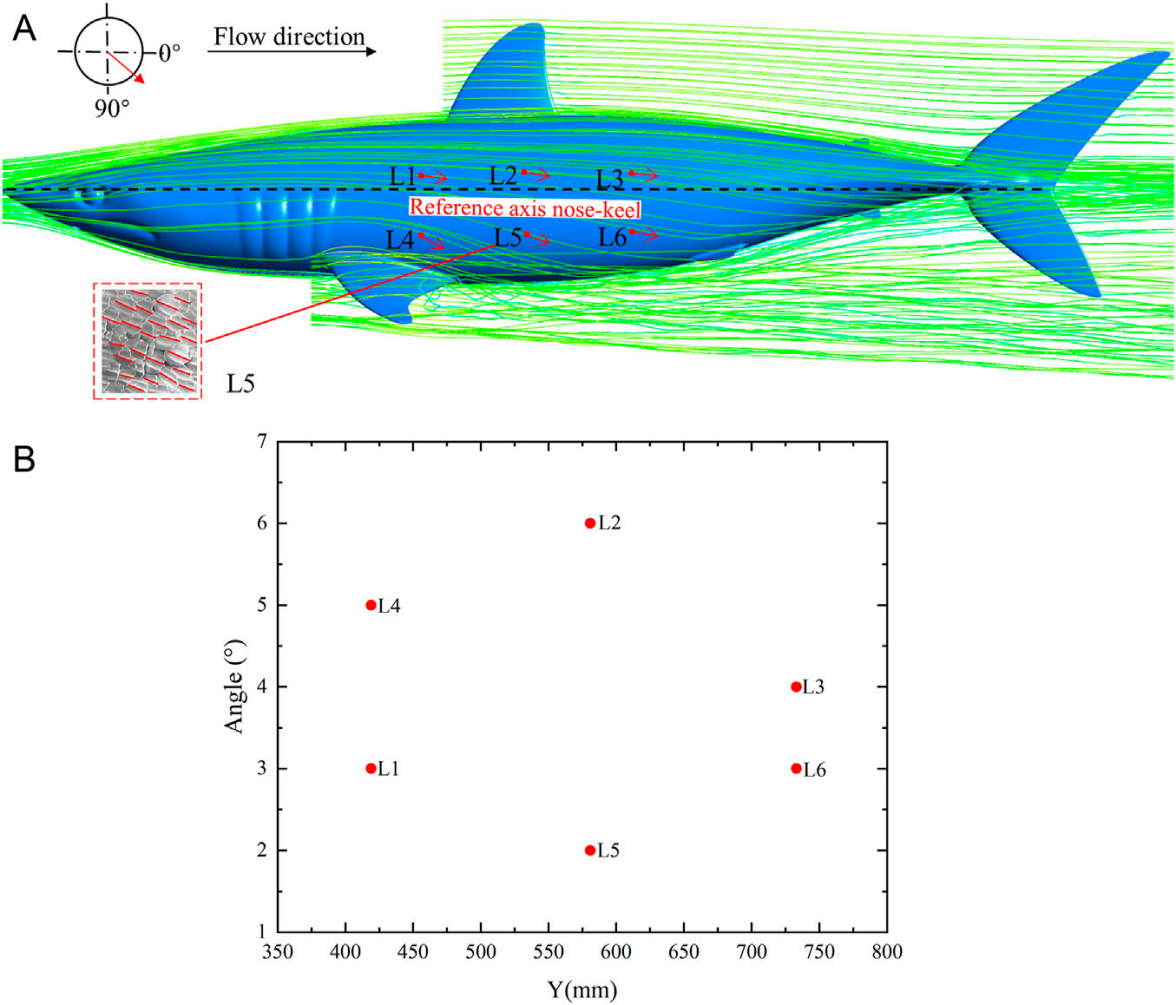
Streamlines of the flow field around the shark and the scale riblets angle along the flow direction in the cruising attitude. **(A)** Streamlines of the flow field and the orientation of angles, **(B)** The values of the angles. The arrows indicate the angle of scale riblets in relation to the streamline as shown in the upper left scheme. The trend of the scale riblet of sample L5 is shown in the lower left corner. The longitudinal reference axis is from nose to caudal fin keel of the shortfin mako shark.

Analyzing the relative relationship between scale direction and flow field has a certain significance as a reference for arranging riblet surface according to flow field characteristics in engineering. Generally, the swing range of shark body is relatively small at cruising speed (Porter et al., 2011). In the cruising attitude, streamlines of the flow field around the shortfin mako shark and the scale riblets angle along the flow direction, as shown in Figure 7. The flow direction is referenced according to the longitudinal axis of the shark, passing from the nose to the caudal fin keel. The streamlines of the flow field changed with the curvature of the shark body, and the angles between the scale riblets and the streamlines of the shortfin mako shark were generally oriented with the flow (Figure 7A). The angles (Supplementary Table 3) were slightly variable on the lateral surface of shark body, with the values of angles ranging from 2° (L5) to 6° (L2) (Figure 7B).

**FIGURE 8:**
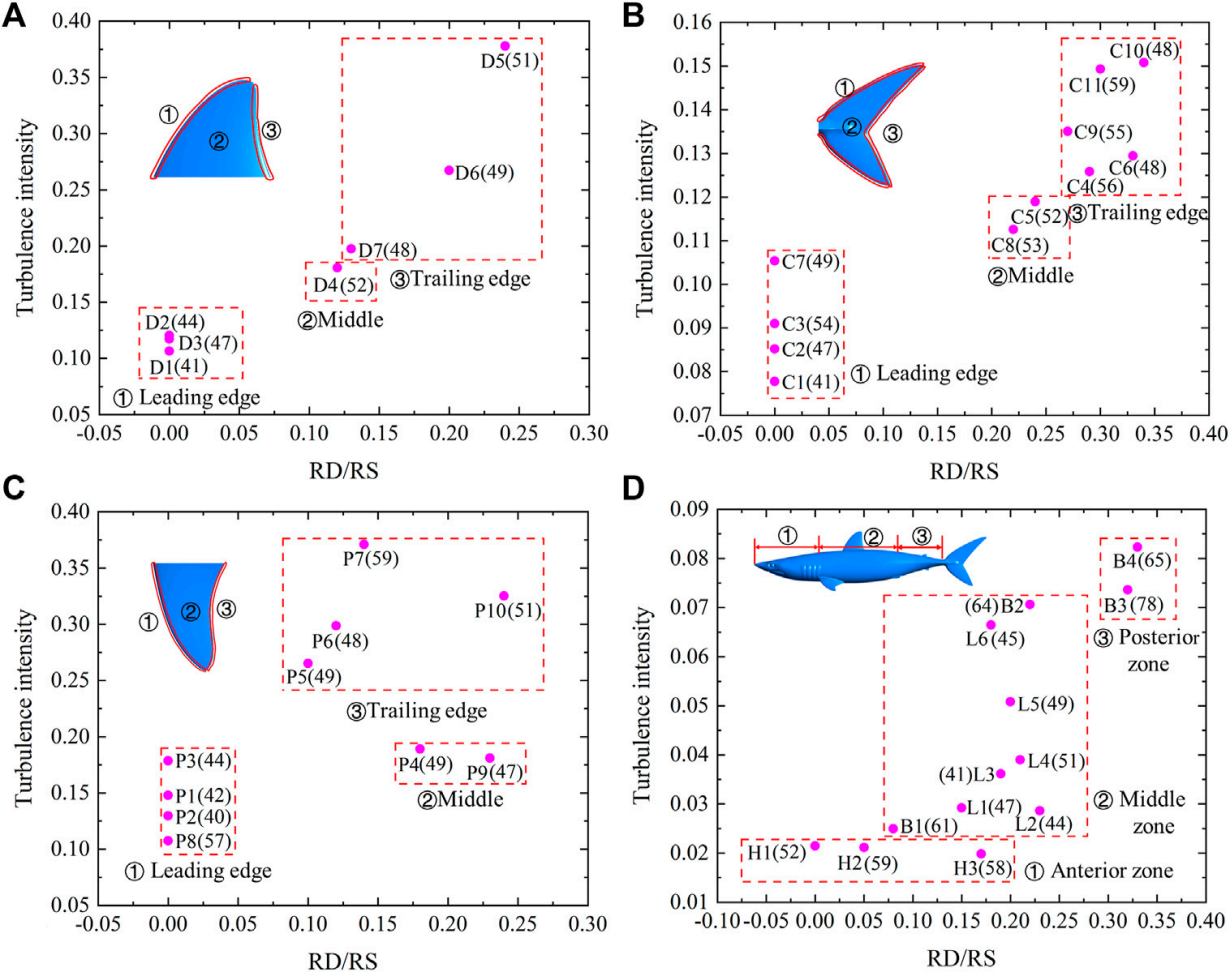
Relationship between the turbulence intensity and riblets RD/RS at y + = 20. **(A)** First dorsal fin, **(B)** Caudal fin, **(C)** Pectoral fin, **(D)** Shark body. Each pink dot represents an independent sample. The numbers in brackets indicate the density of shark skin scales. The serial numbers represent different areas of the shark.

The relationship between the turbulence intensity and the riblets RD/RS at y + = 20 is shown in Figure 8 (Supplementary Table 2). The riblet RD/RS and turbulence intensity gradually increased along the leading, middle, and trailing edges of the first dorsal fin (Figure 8A). The scales on the leading edge of the first dorsal fin were almost smooth, and the riblet RD/RS was 0. The turbulence intensity at the leading edge of the first dorsal fin was 0.106–0.121. The RD/RS in the middle area was 0.12, and the turbulence intensity was 0.181. The RD/RS of the trailing edge was 0.13–0.24, and the turbulence intensity was 0.198–0.378. The density of the middle area was larger than in the other locations. The riblets RD/RS and turbulence intensity progressively increased along the leading, middle, and trailing edges of the caudal fin (Figure 8B). The riblet RD/RS on the leading edge of the caudal fin was 0, and the turbulence intensity of the leading edge of the caudal fin was 0.078–0.105. The riblet RD/RS and turbulence intensity in the middle area were 0.22–0.24 and 0.112–0.119, respectively. The riblet RD/RS of the trailing edge was 0.27–0.34, and the turbulence intensity was 0.126–0.151. The density also increased along the leading, middle, and trailing edges, but they were smaller in samples C6 and C10. The turbulence intensity of the pectoral fin increased along the leading, middle, and trailing edges (Figure 8C). The scale riblets of leading edge were not prominent, the RD/RS ratio was 0, and the turbulence intensity was 0.107–0.179. The turbulence intensity at the middle and trailing edges were 0.181–0.189 and 0.265–0.371, respectively. The scale riblets of the middle and trailing edges of the pectoral fin were found, and the ratio of RD/RS was 0.1–0.24. The maximum density of 59 was found in sample P7. It can be seen that the riblet RD/RS and turbulence intensity increased gradually from the anterior zone, middle zone, to the posterior zone (Figure 8D). In the anterior zone, the ranges of RD/RS and turbulence intensity were 0.05–0.17 and 0.019–0.021, respectively. The middle zone of shark included the back side and lateral surface, and it had RD/RS values of 0.08–0.23, and turbulence intensities of 0.025–0.071. The densities of scales in the middle zone of the shark body were smaller than other surfaces. The RD/RS and turbulence intensity of the posterior zone of the shark body were 0.32–0.33 and 0.074–0.082, respectively. Sample B3 had the maximum density of 78.

The most inconspicuous riblet structures of scales were found on the leading edges of the shark fins and nose, which allowed us to speculate that the pressure and wall shear stress at these locations may be the physical reasons underlying this tendency. The scale riblet RD/RS values were close to 0 where the pressure or wall shear stress was large (Table 3). For instance, the pressure was high on the leading edge of the pectoral fin (P8), dorsal fin (D1, D2), caudal fin (C1, C7), and the nose (H1). The wall shear stress at these locations, i.e., C2, C3, D3, P1, P2, and P3, was also very high, although the corresponding pressures were smaller.

**TABLE 3:** The pressure, wall shear stress (wss) and RD/RS of shark skin scales.

Nose and the leading edge of shortfin mako shark fins												
Region	P8	D1	D2	C1	C7	H1	C2	C3	D3	P1	P2	P3
Pressure (Pa)	11081	10183	6784	6447	7174	9626	3089	1353	2675	−5306	−9860	−3565
WSS (Pa)	45	82	136	126	90	88	137	212	228	296	258	220
RD/RS	0	0	0	0	0	0	0	0	0	0	0	0
Lateral side of shortfin mako shark												
Region	L1	L2	L3	L4	L5	L6	—	—	—	—	—	—
Pressure (Pa)	−1604	−1431	−2037	−3614	−1606	−2523	—	—	—	—	—	—
WSS (Pa)	14	14	15	20	13	13	—	—	—	—	—	—
RD/RS	0.15	0.23	0.19	0.21	0.2	0.18	—	—	—	—	—	—
Back side of shortfin mako shark												
Region	H2	B1	B2	B3	B4	—	—	—	—	—	—	—
Pressure (Pa)	−445	−1281	−1738	−535	1546	—	—	—	—	—	—	—
WSS (Pa)	18	17	15	13	11	—	—	—	—	—	—	—
RD/RS	0.05	0.08	0.22	0.32	0.33	—	—	—	—	—	—	—
Ventral side of shortfin mako shark												
Region	V1	V2	V3	—	—	—	—	—	—	—	—	—
Pressure (Pa)	1135	−1063	−1275	—	—	—	—	—	—	—	—	—
WSS (Pa)	10	6	12	—	—	—	—	—	—	—	—	—
RD/RS	0.36	0.26	0.33	—	—	—	—	—	—	—	—	—

The riblet structures were found on the surface of the scales where the pressure and wall shear stress are smaller. The riblet RD/RS values on the lateral side of shark were 0.15–0.23, and the ranges of pressure and wall shear stress were relatively small. The larger the riblet RD/RS, the smaller the wall shear stress was on the back of the shark. The riblet RD/RS progressively increased along the back of the shark, with the ratios of 0.05, 0.08, 0.22, 0.32, and 0.33 from anterior to posterior. Along the same span, the wall shear stress gradually decreased, with values of 18, 17, 15, 13, and 11 Pa, respectively. The riblets RD/RS on the ventral side of the shark (V1, V2, and V3) were large, and their pressures and wall shear stresses were small.

## Discussion

The differences in shark surface morphology at different positions on the shark body have been described in many previous studies. Reif and Dinkelacker found that the scales on the dorsal side of the shark (*Isurus oxyrinchus*) were relatively wide, while the scales on trailing edge of fins were narrow (Reif and Dinkelacker, 1982). The present study confirmed their results. Díez et al. characterized the parameters of skin scales along the body of a shortfin mako shark (Díez et al., 2015). Compared with the present study, they focused on the effects of scale riblet height on the hydrodynamic characteristics of shark, but we pay more attention to the ratio of riblet depth to spacing, and try to find the relationship between the parameters of the macro main flow field and the morphology, riblet size, and angle of scales in different body regions. Sharks have evolved optimized surface structures that adapt to their surroundings in order to maximize their survival. Here, it was found that the shark skin scales with inconspicuous microstructure are located in regions with a laminar boundary layer, and scales with larger riblet RD/RS are located in regions with a turbulent boundary layer. The microstructural features of shark skin can be reasonably explained using previous research on the distribution of the drag on the actual surface, which indicated that the skin friction coefficient in turbulent regions are much larger than in laminar regions (Zhou et al., 2020).Therefore, it can be concluded that bionic riblet surfaces would be most effective for drag reduction in areas with fully developed turbulence.

Normally, the surface of a workpiece is fully covered with microstructural grooves for drag reduction. However, this may have a negative effect when the microstructure is placed in regions that experience the laminar flow. The results of this study show that it is not necessary to use the microstructural grooves to reduce drag in regions of a workpiece that have laminar flow, and microstructural grooves of suitable size should be integrated in turbulent flow regions, where they will have a greater drag reduction effect. This work provides theoretical support for the application of bionic riblet surfaces in engineering practices.

## Data Availability

The original contributions presented in the study are included in the article/Supplementary Material, further inquiries can be directed to the corresponding author.
