# Supplementary figures and images for: Relationship Between Skin Scales and the Main Flow Field Around the Shortfin Mako Shark Isurus oxyrinchus
Source: Front Bioeng Biotechnol. 2022 Apr 25;10:742437. doi: 10.3389/fbioe.2022.742437 (PMC9081372; doi:10.3389/fbioe.2022.742437)

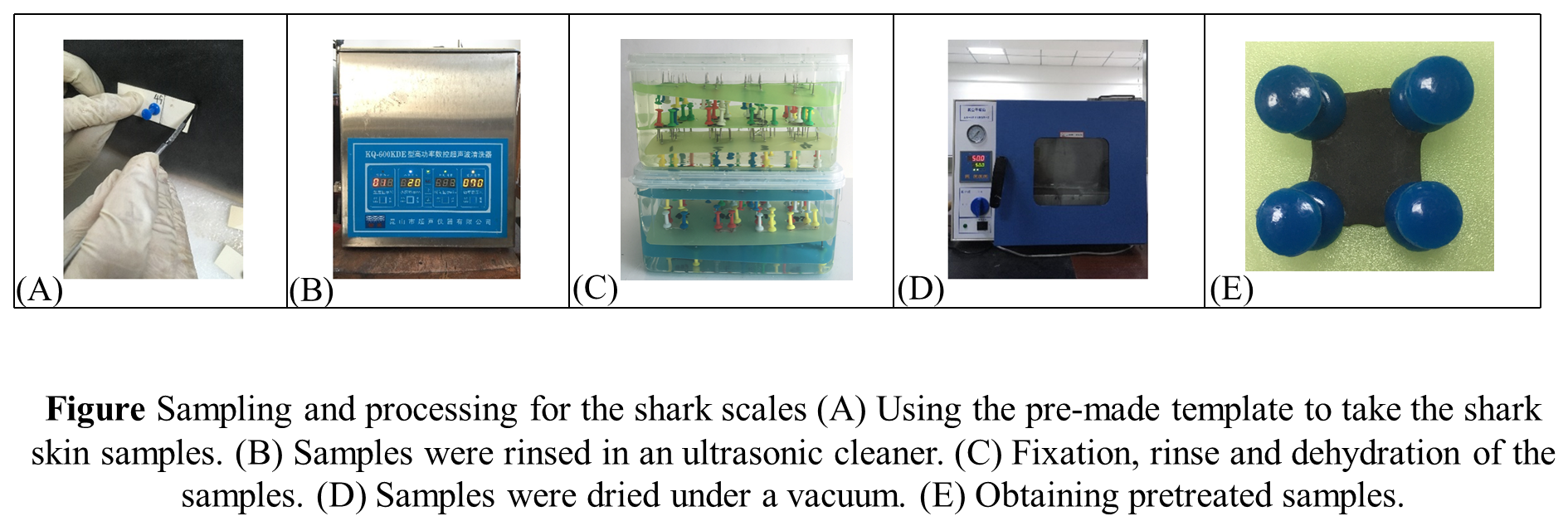

Supplement: Supplementary file 3 [file Image1.TIF]
